# Clinical relevance and validity of TLICS system for thoracolumbar spine injury

**DOI:** 10.1038/s41598-020-76473-9

**Published:** 2020-11-11

**Authors:** Chan-Jin Park, Sung-Kyu Kim, Tae-Min Lee, Eric T. Park

**Affiliations:** 1 Department of Orthopaedic Surgery, Chonnam National University Medical School and Hospital, 8 Hakdong, Donggu, Gwangju, 61469 Republic of Korea; 2 Department of Orthopaedic Surgery, Emory Spine Center, Emory University, Atlanta, USA; 3 Department of Biology, College of Arts and Sciences, Emory University, Atlanta, USA

**Keywords:** Medical research, Risk factors

## Abstract

In order to enhance the reliability of the application to clinical practice of the TLICS classification, we retrospectively reviewed the patients with thoracolumbar spine injuries who underwent magnetic resonance imaging (MRI) and analyzed the validity of the TLICS classification and the necessity of MRI. We enrolled 328 patients with thoracolumbar spine injury who underwent MRI. All patients were classified into conservative and operative treatment groups. The TLICS score of each group was analyzed and the degree of consistent with the recommended treatment through the TLICS classification was examined. Of the total 328 patients, 138 patients were treated conservatively and 190 patients were treated by surgery. Of the 138 patients who underwent conservative treatment, 131 patients (94.9%) had a TLICS score of 4 points or less, and matched with the recommendation score for conservative treatment according to the TLICS classification (match rate 94.9%, 131/138). Of the 190 patients who underwent operative treatment, 160 patients (84.2%) had a TLICS score of 4 points or more (match rate 84.2%, 160/190). All of 30 mismatched patients with a TLICS score of 3 points or less (15.8%) had stable burst fracture without neurological deficit. We retrospectively reviewed the validity of the TLICS classification for the injuries of the thoracolumbar spine, based on MRI in a large group of patients. Treatment with TLICS classification showed high validity, especially in conservative group, and MRI should be an essential diagnostic tool for accurate evaluation of posterior ligamentous complex injury.

## Introduction

Thoracolumbar injury is usually caused by high energy injuries and the possibility of neurological deficits due to spinal cord injury is always present. In particular, the thoracolumbar junction is a mechanically contradictory section between the movable lumbar and relatively non-movable thoracic regions, and thoracolumbar spine injury accounts for the majority of total spinal injuries and the prevalence of injury is higher than that of other parts^1^. Therefore, the precise diagnosis and proper treatment of thoracolumbar injuries are important. Despite the many studies on thoracolumbar injuries, there has been controversy in its classification and treatment. There have been several classifications of thoracolumbar injuries such as the Denis, McAfee, and AO-Magerl classifications. However, their reliability and validity remain controversial because most previous classifications focused only on structural characteristics, but did not reflect neurological deficits and did not provide general guidelines for determining treatment methods. Thus, a new classification is necessary^1,2^.

In 2005, Vaccaro et al.^3^ presented a new classification called Thoracolumbar Injury Classification and Severity Score (TLICS) based on three factors: the morphology of injury, the integrity of the posterior ligamentous complex, and the neurologic status. Many studies have shown that the TLICS classification has a relatively high reliability and validity, and it can be useful to select the adequate treatment plan as well as to classify the mechanism of injury^4–6^. However, in order to enhance the reliability of the application to clinical practice, it is necessary to evaluate the validity of the TLICS classification through a large patient study.

In this study, we retrospectively reviewed the patients with thoracolumbar spine injuries who underwent magnetic resonance imaging (MRI) and discussed the validity of the TLICS classification by investigating the correlation between treatment protocol according to the TLICS classification and the treatment methods that were used actually.

## Materials and methods

This report and protocols were approved by the Institutional Review Board of Chonnam National University Hospital. The patients were informed that we wanted to submit data for publication, and informed consent was obtained from all patients. Also, all methods were carried out in accordance with relevant guidelines and regulations.

**Figure 1:**
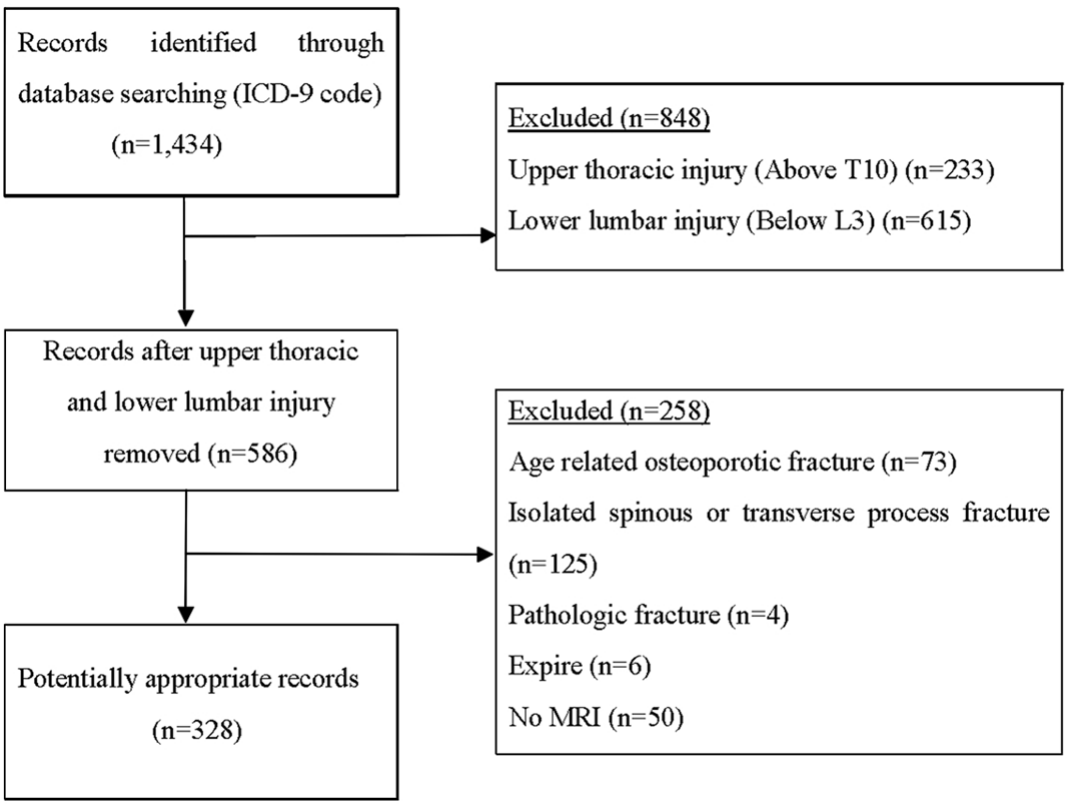
Flow diagram indicating inclusion and exclusion criteria.

We studied patients with thoracolumbar spine injury who visited our hospital from 2000 to 2016. Among the 1434 registered patients with the International Code of Disease Control (ICD-9) of thoracic injuries, lumbar injuries, and spinal cord injuries in the medical information system, 586 patients were selected except for those patients who had injuries above the 10th thoracic vertebrae or below 3th lumbar vertebrae. Among the 586 patients, 328 patients were selected for the study excluding pathologic fractures (infection or tumor), minor injuries such as spinous process fractures and transverse fractures, age-related injuries such as osteoporotic compression fractures due to a simple fall, and patients who did not have MRI scan (Fig. 1). The cause of injuries and neurological deficits at the time of injury were examined. The degree of neurological deficits was measured by the American Spinal Injury Association (ASIA) impairment scale. All patients underwent plain radiography, computed tomography (CT), and MRI of the thoracolumbar spine. In thoracolumbar fractures or TLICS classification, MRI is not an essential tool for diagnosis, and it is usually performed at the surgeon’s discretion. However, in our study, patients who did not have MRI scan were excluded to confirm the validity of TLICS classification more clearly. At the time of treatment, the treatment method was determined according to surgeon’s judgement by referring to MRI, not the TLICS classification. Overall, 328 patients were classified into conservative and operative treatment groups. Indication for operative treatment include burst fracture with fifteen degrees or greater kyphosis, loss of more than 50% of anterior vertebral body height, more than 25% of the canal compromised by retropulsed fragment and neurologic deficit in the presence of spinal cord compression^7^.

The TLICS score of each group was analyzed retrospectively during the study and the degree of agreement with the recommended treatment through the TLICS classification was examined. The TLICS classification was determined by summing the scores of the three factors, including the morphology of injury, the integrity of the posterior ligamentous complex, and the neurologic status. If the total score is 3 points or less, a conservative treatment was considered. If the total score was 5 points or more, a surgical treatment was considered. If the total score was 4 points, the treatment method was decided according to the surgeon’s discretion. Similar to Vaccaro et al.^3^, the injury to the posterior ligamentous complex of our study was assessed by widening of the interspinous space, diastasis of the facet joints, facet perch or subluxation, and vertebral body translation or rotation shown in plain radiography, CT, and MRI. In addition, if a tear of the posterior ligamentous complex was clearly observed on MRI scan, it was assessed as a definite disruption and if there was no clear tear of the posterior ligamentous complex but a signal change on the MRI was observed, it was assessed as a suspected/indeterminate disruption.

## Results

Of the total 328 patients, 192 patients were male and 136 were female. The mean age was 50.6 years (21 to 86) and the average follow-up duration was 88.6 weeks (36 to 693). Of the total 328 patients, 138 patients (42.1%) were treated conservatively and 190 patients (57.9%) were treated by surgery. There were 232 cases (70.7%) by falls, 79 cases (24.1%) by traffic accidents, and 17 cases (5.2%) by direct external forces (Table 1).

**Table 1:** Patient demographics.

	Conservative treatment (n = 138)	Operative treatment (n = 190)
**Mean age (years)**	58.3 (23–86)	45.0 (21–75)
**Cause of injury (%)**		
Fall	91 (65.9%)	141 (74.2%)
Traffic accident	41 (29.7%)	38 (20.0%)
Direct injury	6 (4.4%)	11 (5.8%)
**Follow up duration (weeks)**	71.2 (36–331)	102.0 (53–693)

### Initial conservative treatment group

**Figure 2:**
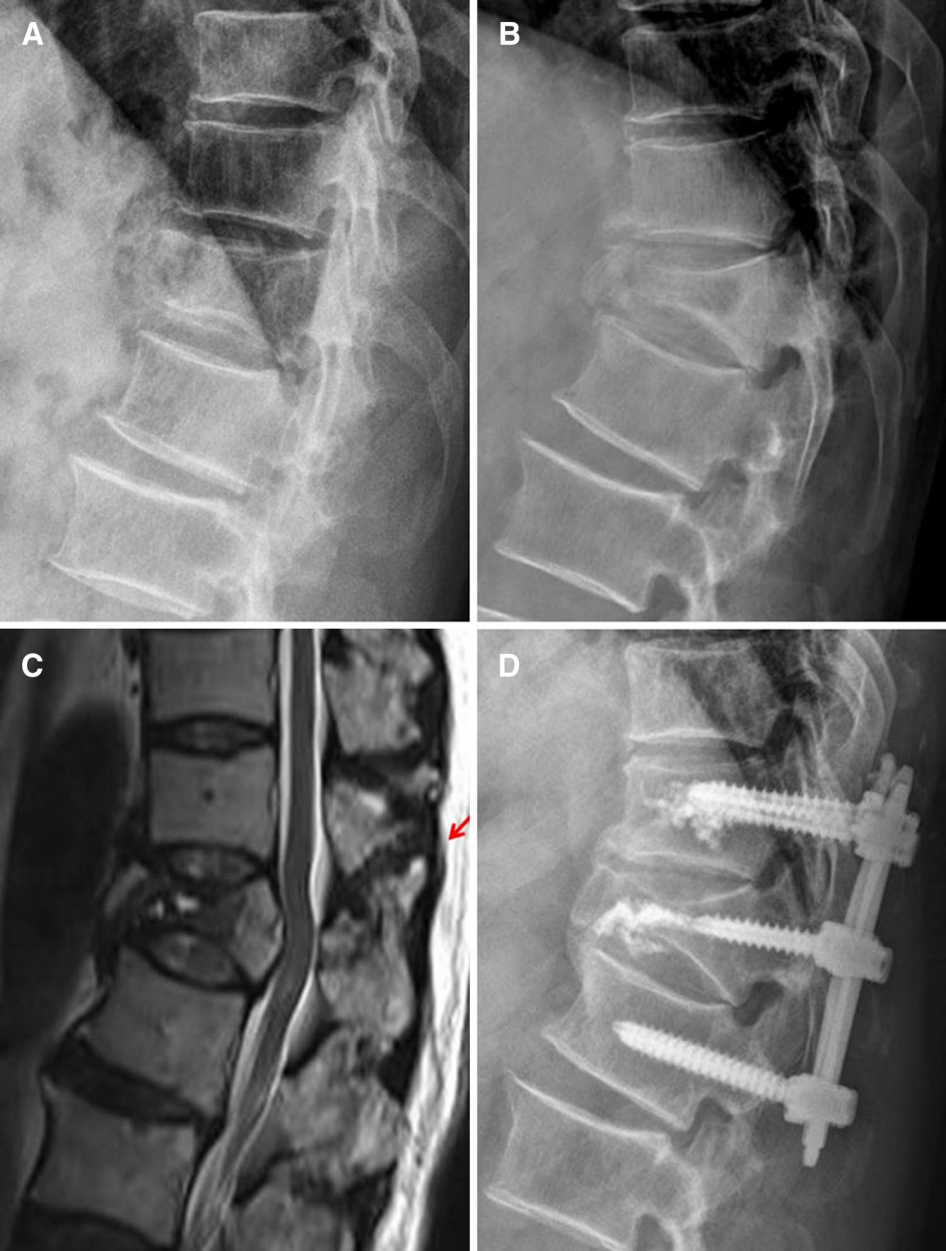
(**A**) A 64-year-old male patient was initially diagnosed with T12 stable burst fracture. (**B**) Kyphosis increased along with a collapse of the vertebral body after 2 weeks. (**C**) MRI showed further collapse of the vertebral body and occult injury of posterior ligamentous complex (red arrow). (**d**) At three weeks after the initial injury, patient underwent posterior fixation.

The mean age of patients who underwent conservative treatment was 58.3 years (23 to 86). The most common cause of injury was falling in 91 patients, followed by traffic accidents (41 patients), and direct injuries (6 patients). Neurologic status at the time of injury was ASIA scale E in 134 patients (97.1%), ASIA scale D in 2 patients (1.45%), and ASIA scale C in 2 patients (1.45%). The mean follow-up period was 71.2 weeks (36 to 331). Patients who underwent conservative treatment with the thoracolumbosacral orthosis (TLSO) for at least 6 weeks were allowed to mobilize after pain relief. The TLICS score was assessed retrospectively. The mean TLICS score of the patients who underwent conservative treatment was 2.06 points (1 to 7) and TLICS score in 7 patients was 5 points or more. Of the 138 patients who underwent conservative treatment, 131 patients (94.9%) had a TLICS score of 4 points or less, and matched with the recommendation score for conservative treatment according to the TLICS classification (Table 2). Among them, 18 patients (13.0%) had a TLICS score of 4 points (TLICS 4). One of the 7 mismatched patients had a conservative treatment due to very poor general conditions, although it was determined that surgery was initially necessary. Six of the 7 mismatched patients were initially diagnosed with stable burst fractures (TLICS score, 2 points). Later, it was revealed through a re-evaluation that they had distraction injuries, and all patients underwent delayed surgery (Fig. 2). Of the patients who underwent conservative treatment, 10 patients (7.2%) failed conservative treatment and eventually had an operation at an average 46 days (30 to 159) after the injury. As mentioned above, 6 patients were misdiagnosed with stable burst fractures and the other 4 patients had TLICS scores less than 4 points. However, during the follow-up period, the vertebral body of two patients collapsed more with lower back pain and one patient suffered uncontrolled lower back pain. One patient developed a tingling sensation in legs that did not exist at the time of injury. None of the patients with TLICS 4 had treatment failure.

**Table 2:** TLICS score of the patients and match rate with the recommendation score according to the TLICS classification

TLICS score	Conservative treatment (n = 138)	Operative treatment (n = 190)
1 ~ 3	113 (81.9%)	30 (15.8%)
4	18 (13.0%)	25 (13.2%)
> 5	7 (5.1%)	135 (71.0%)
**Matched**	131 (94.9%)	160 (84.2%)
**Mismatched**	7 (5.1%)	30 (15.8%)

### Operative treatment group

**Figure 3:**
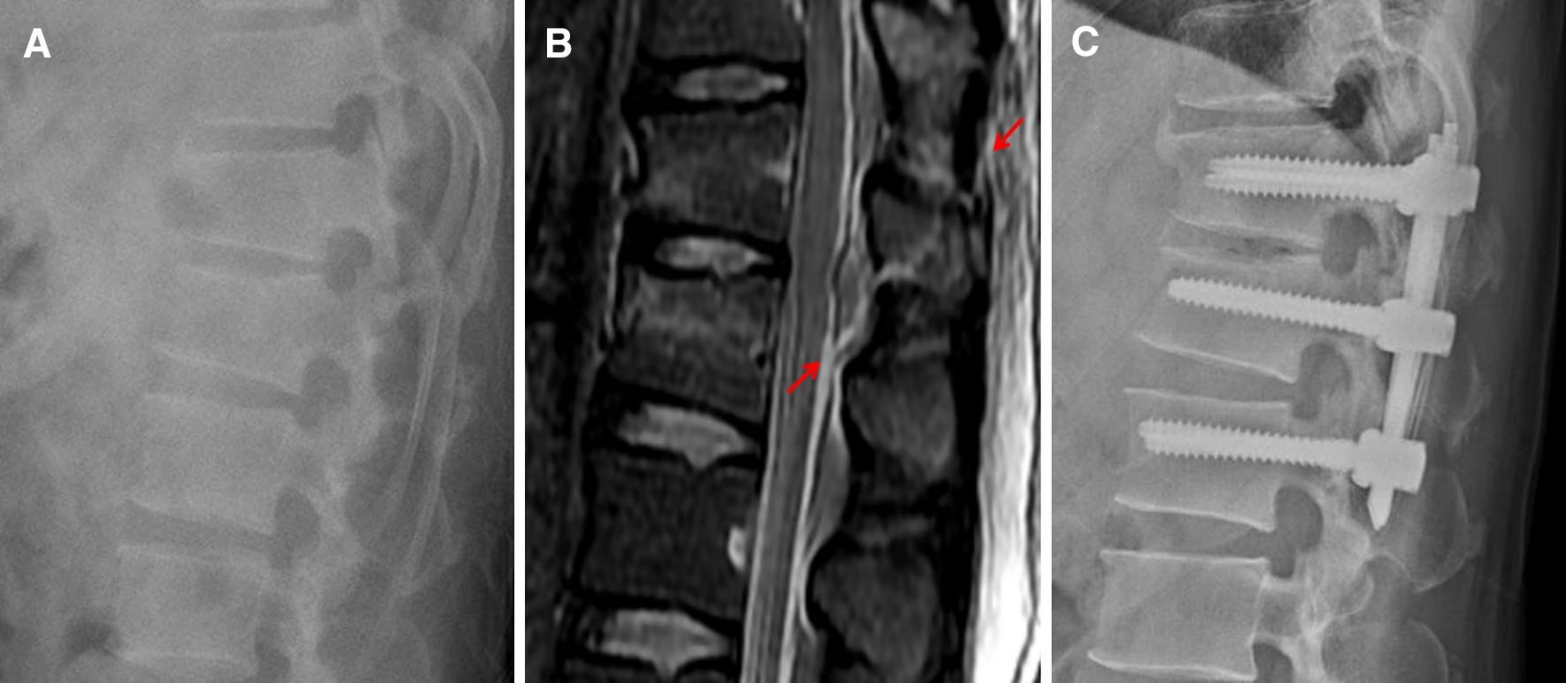
(**A**) A 46-year-old male patient underwent conservative treatment initially because the diagnosis was considered as L1 stable burst fracture on plain radiography. (**B**) The patient complained of more severe pain after 2 days and MRI was performed. MRI showed occult injury of posterior ligamentous complex (red arrow). (**C**) The patient underwent posterior fixation and plain radiography at postoperative 29 months showed well maintained vertebral height.

The mean age of patients who underwent operative treatment was 45.0 years (21 to 75). The most common cause of injury was falling in 141 patients, followed by traffic accidents and direct injuries. Neurologic status at the time of injury was ASIA scale E in 96 patients (50.5%), ASIA scale D in 20 patients (10.5%), ASIA scale C in 30 patients (15.8%), ASIA scale B in 11 patients (5.8%), and ASIA scale A in 33 patients (17.4%). The mean follow-up period was 102.0 weeks (53 to 693). The mean TLICS score of the patients who underwent operative treatment was 5.97 points (2 to 10). Of the 190 patients who underwent operative treatment, 160 patients (84.2%) had a TLICS score of 4 points or more, and matched with the recommendation score for operative treatment according to the TLICS classification (Table 2; Fig. 3). Among them, 25 patients (13.2%) had TLICS 4. All of 30 patients with a TLICS score of 3 points or less (15.8%) had stable burst fracture without neurological deficit (TLICS score, 2 points). Postoperative complications occurred in 10 patients (5.3%). Six patients with fixation failure and 1 patient with pseudoarthrosis underwent re-operations. Three patients with infection also underwent additional operation including debridement. None of the patients with TLICS 4 had treatment failure or complication.

## Discussion

The classification of thoracolumbar spine injury was first described by Bohler in 1929 and then was rapidly developed by Denis' Three Column Theory in 1983. After that, McAfee classified burst fractures in the Three Column Theory as stable and unstable fractures. There have been many other classifications for thoracolumbar spine injuries, but there have been no classifications that provided general guidelines for determining treatment methods until recently. However, Vaccaro introduced the TLICS classification in 2005^3^, focusing on neurological deficits and posterior ligamentous complex injuries to overcome the weaknesses of previous classifications. It has evolved from a classification for morphological and structural diagnosis to a classification for adequate treatment plan. From that point on, some studies on the validity of the TLICS classification have been performed^8–10^. But most of them have been limited by the small number of patients, or whole thoracic and lumbar spine injuries not just thoracolumbar junction^4,5,11^. Also, diagnostic methods for thoracolumbar injuries were mixed with plain radiograph, CT, and MRI. Therefore, we analyzed the TLICS classification using MRI for a large number of patients with injuries limited to the thoracolumbar junction.

As a result, we found that there was a significant correlation between the treatment method selected by clinical practice and the treatment method according to the score of the TLICS classification. Of the 138 patients who underwent conservative treatment, 131 patients (94.9%) had 4 points or less, which was recommended for conservative treatment in the TLICS classification. Furthermore, in the 7 patients (5.1%) who had more than 5 points, one patient was in an inoperable state with poor general condition, while the other six patients who were misdiagnosed with stable burst fracture at the time of injury should have been in the operative group according to TLICS score if they were correctly diagnosed initially. In retrospect, mismatched 7 patients were actually belong to the operative group. Overall, the concordance rate of the TLICS classification in the conservative treatment group was 100% (131/131 patients). Of the patients who underwent conservative treatment, 128 patients (92.8%) had successful clinical outcomes until the end of follow-up. But the other 10 patients (7.2%) who failed the conservative treatment underwent delayed surgeries. However, excluding the 6 patients who were misdiagnosed with stable burst fracture, only 4 patients in the conservative group (131 patients) actually underwent delayed surgeries. Therefore, when judging based on the correct application of the TLICS classification, the success rate of the conservative treatment was about 97%.

Of the 190 patients who underwent operative treatment, 160 patients (84.2%) had 4 points or more, which was recommended for operative treatment in the TLICS classification. Particularly noteworthy was the fact that 30 mismatched patients (15.8%) who had less than 3 points were all diagnosed with stable burst fractures without neurologic deficits, which represents 2 points in the TLICS classification. In previous studies, an appropriate treatment method for stable burst fractures has been controversial. A prospective randomized controlled study of patients with stable thoracolumbar burst fractures with a mean follow-up of 4.3 years by Siebenga et al.^12^ showed statistically significant improvements in terms of pain relief and function in groups treated surgically. Wood et al.^13^ performed a prospective study of stable burst fractures of the thoracolumbar spine in 2003. Twenty-three of the 47 patients were treated conservatively and the other 24 patients were treated surgically. There was no statistically significant difference between the two groups with respect to their ability to return to daily life activities or pain at last follow-up. However, in the 18 years long-term follow-up study by the same authors in 2015^14^, they reported that the conservative group (18 patients) showed statistical superiority in terms of pain and function compared to the operative group (19 patients). Therefore, Wood et al.^1,4^ reported that surgical treatments had no significant advantage over conservative treatments in patients with thoracolumbar stable burst fracture without neurological deficits. In the TLICS classification, stable burst fractures without neurologic deficits accounts for two points and conservative treatment is recommended. However as mentioned above, conservative treatment and operative treatment are controversial in patients with stable burst fracture. Mostly, it is determined by the surgeon’s preference. In this study, we found that it was not easy to determine consistent treatment methods in the past, which did not consider the treatment guidelines according to the TLICS classification, and the preference of the surgeon has a great influence on the treatment plan in our institute. However, it is not an easy decision at present. In addition, it is more difficult to determine the treatment plan of unstable burst fractures without neurologic deficits (TLICS score, 4 points). With TLICS 4, the decision of treatment method is completely decided by the surgeon. However, its biggest disadvantage is that accurate decision-making is difficult when choosing the appropriate treatment for TLICS 4 thoracolumbar fractures. The treatment of patients with TLICS 4 is currently the most important and focused^15,16^. Although many studies point to the fact that both operative and conservative treatments yield similar clinical outcomes in patients with TLICS 4 fractures, some studies show that the clinical outcomes vary according to the treatment selected^5,12–14^. In our study, the percentage of TLICS 4 was 13% in the conservative treatment group and 13.2% in the operative treatment group. There were no treatment failures or complications in patients with a TLICS 4 in both groups. However, we do not believe that this result support the paper of Vaccaro et al.^3^ that the treatment method of TLICS 4 was decided according to the surgeon’s discretion. Further research is needed in a larger number of patients, especially for patients with TLICS 4 such as unstable burst fractures without neurologic deficits.

Joaquim et al.^17^ performed a retrospective study on the validity of the TLICS classification in 458 patients with thoracolumbar spine injury. In this study, 310 patients with conservative treatment and 148 patients with surgical treatment were retrospectively compared with the TLICS classification. Patients who underwent conservative treatment were almost matched (99%) with the treatment according to the TLICS scores. However, there was only partial consistency (46.6%) in the surgical treatment group. In the study by Joaquim et al., MRI was not performed on all patients, but performed according to the surgeon’s discretion at the time of the injury. Furthermore, upper thoracic and lower lumbar injuries were included in addition to thoracolumbar junction injuries. In our study, MRI was performed in all patients and only injuries of thoracolumbar junction were included. Of the 190 patients who underwent operative treatment, 160 patients had TLICS score of 4 or more, which was much more matched (84.2%) than other studies. Because MRI was performed on all patients, a diagnosis of a suspected injury of the posterior ligamentous complex was much more possible than that of other studies^18^ (Fig. 3). For this reason, the TLICS score is elevated in patients treated surgically and in conclusion, the consistency of the TLICS classification was increased. In our study, the presence of a posterior ligamentous complex injury was an important factor in the selection of operation, even when determining the treatment method without considering the TLICS classification^5,10,17,19^. Of course, the TLICS classification has high reliability and validity in determining treatment methods. In addition to this, the effectiveness of the TLICS classification will be higher if the MRI is checked to confirm posterior ligamentous complex injury more accurately. Therefore, MRI should be an essential procedure rather than an option in order to determine the appropriate treatment for thoracolumbar fractures. Although the evaluation of injury to the posterior ligamentous complex was somewhat ambiguous in a study by Vaccaro et al.^3^, our study suggests that early MRI examination can be a decisive tool for determining the treatment method using the TLICS classification.

The limitations of this study are as follows: First, this study was performed retrospectively and we were able to evaluate the validity of the TLICS classification for deciding on treatment methods. However, it was difficult to prove the validity of the clinical outcomes prospectively according to the TLICS classification because patients with high TLICS score that required operation could not be observed without surgery. Second, multi-institute studies are needed because of possibility of biases for treatment selections within a single institute.

## Conclusions

We retrospectively reviewed the validity of the TLICS classification for injuries of the thoracolumbar spine, based on MRI in a large group of patients. Treatment with TLICS classification showed high validity, especially in the conservative treatment group, and MRI should be an essential diagnostic tool for accurate evaluation of posterior ligamentous complex injury. In addition, it has been found that it is still difficult to establish specific treatment methods for burst fractures without neurological deficit. Therefore, further studies on the TLICS classification are needed to establish more accurate and safe treatment methods for thoracolumbar injuries.
